# Liposclerosing Myxofibrous Tumor: A Separated Clinical Entity?

**DOI:** 10.3390/diagnostics15050536

**Published:** 2025-02-22

**Authors:** Eva Manuela Pena-Burgos, Gabriela Serra del Carpio, Mar Tapia-Viñe, Julia Suárez-González, Ismael Buño, Eduardo Ortiz-Cruz, Jose Juan Pozo-Kreilinger

**Affiliations:** 1 Pathology Department, Gregorio Marañón General University Hospital, 28007 Madrid, Spain; 2 Radiology Department, La Paz University Hospital, 28046 Madrid, Spain; 3 Genomics Unit, Gregorio Marañón General University Hospital, Gregorio Marañón Health Research Institute (IiSGM), 28007 Madrid, Spain; 4 Gregorio Marañón Health Research Institute (IiSGM), 28007 Madrid, Spain; 5 Department of Hematology, Gregorio Marañón General University Hospital, 28007 Madrid, Spain; 6 Department of Cell Biology, School of Medicine, Complutense University of Madrid, 28040 Madrid, Spain; 7 Orthopaedic Surgery and Traumatology Department, La Paz University Hospital, 28046 Madrid, Spain; 8 Pathology Department, La Paz University Hospital, 28046 Madrid, Spain

**Keywords:** liposclerosing myxofibrous tumor, polymorphic fibro-osseous lesions of bone, fibrous dysplasia, bone tumors, femoral tumors

## Abstract

**Introduction**: Liposclerosing myxofibrous tumors (LSMFTs) have been described as an infrequent and peculiar fibrous dysplasia variant with a predilection for the intertrochanteric femoral region and are not globally considered a distinct tumor. Given their features, they may be confused with a variety of entities. Our aim is to analyze the clinical, radiological, histopathological and molecular features of LSMFTs. **Material and Methods**: We report 15 new LSMFT cases managed in our tertiary referral hospital and compare our findings with those of the 241 previous LSMFT cases published in the English medical literature. **Results**: In plain radiography and computerized tomography, LSMFTs are well-defined intraosseous lytic masses with peripheral sclerotic rims and variable amounts of internal calcifications. Histopathologically, LSFMTs consist of variable amounts of spindle cells, bone matrix, adipose tissue, and cystic spaces embedded in a predominantly fibromyxoid stroma. Molecular tests reveal *GNAS* and *TP53* mutations. **Conclusions**: Knowledge of LSMFT and its typical radiological appearance with heterogeneous histopathological findings—especially in small biopsies—are key to preventing the misdiagnosis and overtreatment of affected patients.

## 1. Introduction

Liposclerosing myxofibrous tumors (LSMFTs) are known as a benign fibro-osseous lesion with a predilection for the femoral intertrochanteric region [1,2]. They typically affect adults during the fourth decade of life [1]. They have distinctive radiological features [2] and a variety of histopathological findings [1] and may present malignant transformation in some cases [3,4,5]. LSMFT is not globally considered a distinct tumor and is a controversial entity [1,6,7,8]. In the latest WHO classification of Bone and Soft Tissue Tumors (2020), LSMFTs do not have their own chapter and are only mentioned in the fibrous dysplasia chapter [8]. The objective of this article is to present the clinical, radiological, histopathological, and molecular features of LSMFTs; describe their management; and present a review of the related literature.

## 2. Materials and Methods

La Paz University Hospital is a tertiary hospital and one of the referral hospitals for bone and soft tissue tumor management in Spain. Patients with histological diagnosis of fibrous dysplasia or bone infarct of proximal femur, as well as LSFMT of any other bone location during the period from January 1966 to December 2024, were included in this retrospective study. Clinical information and radiological images were obtained from their medical records. All available materials from the pathology files were reviewed, including hematoxylin- and eosin-stained slides, as well as immunohistochemical stains. Tissues were fixed in 10% formalin (24–48 h), embedded in paraffin, and serially sectioned (5 μm thick). Slides were individually evaluated by two expert pathologists in the field, and the final diagnoses were in agreement.

Molecular testing in the most recent non-decalcified cases was completed retrospectively via Next-Generation Sequencing (NGS). Genomic DNA was extracted from fixed and paraffin-embedded tumor tissue (FFPE), according to the manufacturer’s instructions (Qiagen, Hilden, Germany), using the Gene Read DNA FFPE KIT. The quantity and quality of DNA were assessed using a NanoDrop One spectrophotometer (Thermo Scientific, Waltham, MA, USA) and a Qubit 3.0 fluorometer (Thermo Fisher, Waltham, MA, USA). Hybrid-captured targeted libraries were prepared from 50 ng of genomic DNA with a Sophia Custom Solid Tumor Solution gene panel (Sophia Genetics, Lausanne, Switzerland), according to the manufacturer’s protocol. This panel includes relevant regions of 50 genes involved in solid tumor development. Libraries were denatured and paired-end (2 × 150 bp) sequenced on the Illumina MiSeq instrument (Illumina, San Diego, CA, USA). We used the Sophia DDM platform (SOPHIA GENETICS, Lausanne, Switzerland) to analyze single nucleotide variants and small insertions and deletions. FASTQ files were uploaded to the data portal and aligned with the human reference genome (GRCh37/hg19). After annotation in DDM, non-synonymous variants located in exonic or ±1.2 intronic splice regions were retained, and variants with a minor allele frequency < 0.01 (based on ExAC, GnomAD, and 1000 Genomes databases) were selected for the downstream analysis. At present, there is no standardized method to establish the best cut-off point for variant allele frequency. In this sense, we decided to set the percentage at 5% in the FFPE, as there was a high percentage of tumor in these samples, in an attempt to avoid false positives. We used an Integrative Genomics Viewer (Broad Institute, San Diego, CA, USA) to visualize the variants aligned against the reference genome in order to confirm the accuracy of the variant calls by checking for possible strand biases and sequencing errors. Identified variants were annotated using population databases (The Exome Aggregation Consortium (ExAC) and 1000 Genomes) and specific databases (Ensembl; PubMed; ClinVar; Catalogue of Somatic Mutations in Cancer, COSMIC; and The Cancer Genome Atlas, TCGA). Pathogenicity prediction was established with in silico prediction software: Sorting Intolerant from Tolerant (SIFT), Polyphen 2.0, and Mutation Taster (V6.8.0, Sophia DDM platform). Finally, the pathogenicity of variants was designated according to the recommendations of the American College of Medical Genetics and Genomics and the Association for Molecular Pathology [9].

Previous medical literature case reports containing the terms “liposclerosing myxofibrous tumor” and “polymorphic fibro-osseous lesions of bone” in the PubMed database were reviewed and analyzed. Finally, the findings of the cases from our hospital and those described in the literature were compared.

## 3. Results

A total of 12 LSMFTs, 5 bone infarcts, and 26 fibrous dysplasia cases were found in our files. One of the LSMFT cases was excluded, given that it was an external consultation case, and no radiological information was available. Four fibrous dysplasia cases were morphologically re-classified to LSMFT upon histopathological evaluation. No bone infarct cases were re-classified. As a result, 15 cases of LSMFT were finally included in this study (Table 1). Ethics committee approval was not required, as this was a retrospective observational study. Our study population included eight males and seven females (ratio 1:0.9) aged 18 to 72 (mean age of 38). The clinical presentation consisted of local pain in seven cases, pathological fracture in two cases, and incidental detection in six patients. Prior local trauma history was registered in one case. All the tumors appeared in the femur, with 14 cases occurring in the intertrochanteric region and 1 in the distal diaphysis. In all cases, a single lesion was detected: eight on the right side and seven on the left.

**Figure 1:**
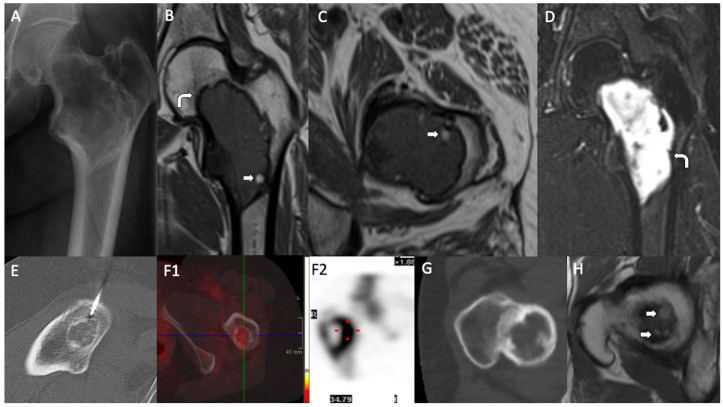
Radiological findings. LSMFTs of three patients. (**A–D**): (**A**) Anteroposterior radiography: geographic lucent lesion with a sclerotic margin and foci of matrix calcification, centered in the intertrochanteric region. (**B**) MIR, coronal and (**C**) MRI, axial T1WI: central medullary lesion that elicits low signal and peripheral hyperintense foci compatible with fat (white arrows). (**D**) MRI, coronal T2WI: high signal. Low-signal sclerotic rim, hypointense on T1WI and T2WI (curve arrows). (**E**,**F**): (**E**) Axial image, CT-guided biopsy: lytic lesion with internal calcified foci. (**F1**) (SPECT) and (**F2**) (bone scintigraphy): moderate and heterogeneous uptake of the lesion (red dot: intense uptake area). (**G**,**H**): (**G**) Axial CT: lytic lesion with a sclerotic rim. (**H**) MRI, axial T1WI: small foci of T1 high signal at the superior aspect of the lesion, suggestive of fat (white arrows).

Plain radiography and computerized tomography (CT) images were available in nine cases. All of these cases presented a similar image, consisting of a well-defined intraosseous lytic mass with a peripheral sclerotic rim. Gross internal calcification was evident in three cases. In two cases, cystic areas with internal trabeculae were observed. No ground-glass matrix was detected. No periosteal reaction, infiltrative pattern, or soft tissue extension were seen. Magnetic resonance images (MRIs) were available in six cases. These images revealed intramedullary lesions, isointense or hypointense to skeletal muscle on T1 weighted images (WI) and hyperintense on T2WI. Peripheral fat areas were found in three lesions. Single-photon emission computed tomography (SPECT) was performed in three cases, displaying variable uptake. In one instance, no uptake was observed; in another, peripheral uptake was noted, and, in another case, moderate and uniform uptake was seen. The considered radiological differential diagnosis included fibrous dysplasia, LSMFT, intraosseous lipoma, aneurysmal bone cyst (ABC), unicameral bone cyst, enchondroma, osteoblastoma, intraosseous ganglion cyst, and chondromyxoid fibroma. Exemplary radiological findings are shown in Figure 1.

**Figure 2:**
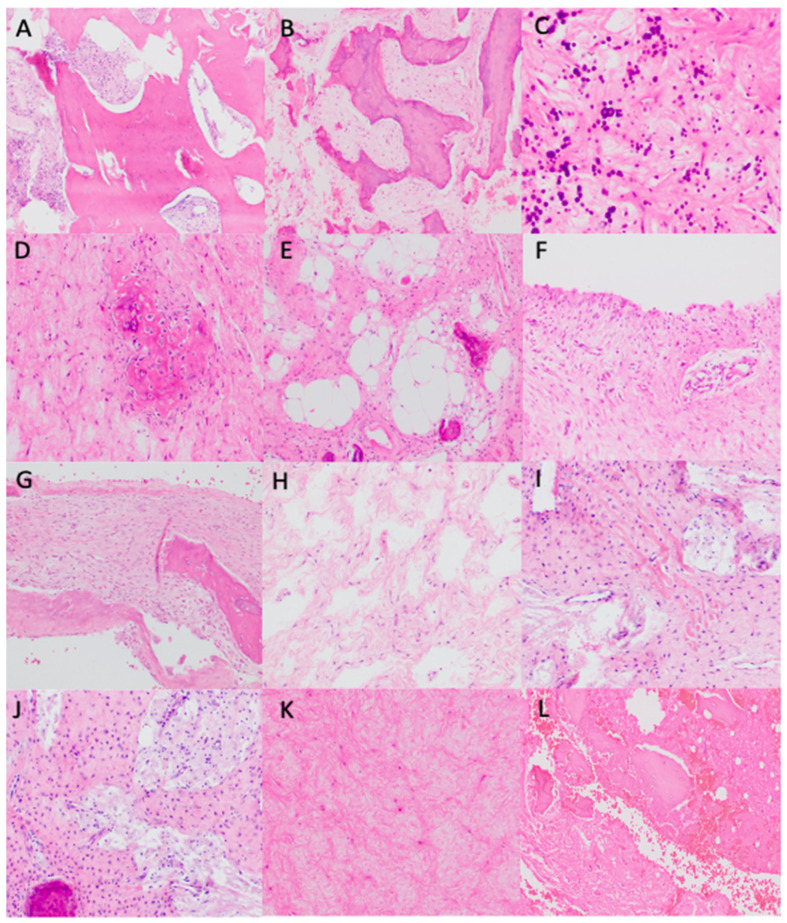
Histopathological findings. (**A**) Sclerotic bone trabeculae (H&E, ×100). (**B**) Pseudopagetoid bone (H&E, ×100). (**C**) Psammomatoid bone (H&E, ×400). (**D**) Immature bone closely related to non-atypical spindle cell in a fibromyxoid stroma (H&E, ×200). (**E**) Adipose tissue intermixed with other components, especially bone-related (H&E, ×200). (**F**) Macrocystic wall without endothelial lining (H&E, ×200). (**G**) ABC-like changes: parallel bone trabeculae to the pseudovascular spaces without endothelial lining (H&E, ×200). (**H**) Microcystic spaces. (**I**) Dense collagen bands in a fibromyxoid stroma (H&E, ×200). (**J**) Xantomized cells intermixed with fibromyxoid stroma and bone (H&E, ×200). (**K**) Hyalinized and fibromyxoid stroma (H&E, ×200). (**L**) Bone infarct area (H&E, ×200).

**Figure 3:**
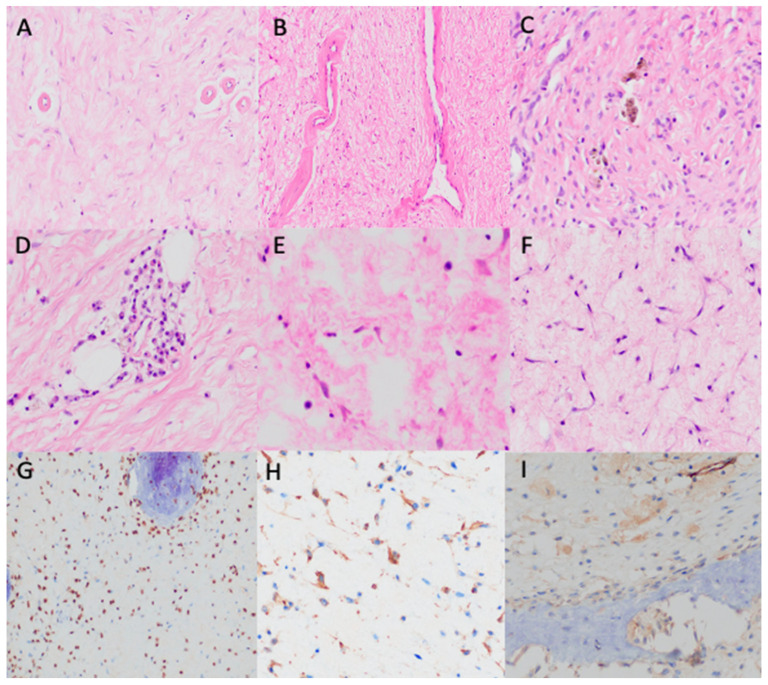
Histopathological findings. (**A**,**B**) Hyalinized vessels (H&E, ×200). (**C**) Hemosiderophages (H&E, ×400). (**D**) Perivascular lymphoplasmacytic infiltrate (H&E, ×400). (**E**) Scattered mast cells (H&E, ×400). (**F**) Stellated and spindled non-atypical cells with scanty cytoplasm and round nuclei (H&E, ×400). (**G**) SATB2 positivity (×400). (**H**) SMA patchy positivity (×400). (**I**) Negative CD34 (×400).

Intralesional resection (curettage) and reconstruction with bone grafting were the selected treatments in 14 cases, with reinforcement via osteosynthesis with a nail plate in 3 of these cases. Wait-and-see management was decided for one patient, due to her poor general condition. The median size of the resected fragments received in the Pathology Department measured 4.4 cm. A pre-surgical percutaneous echo-guided biopsy was performed in two cases with non-typical radiological findings. Histologically, the analyzed tissue was polymorphic. The fibromyxoid stroma was the predominant component in thirteen cases, being more myxoid in two of them. The stroma was loose in one case and more fibrous in another. Dense collagen bands were observed in one case. Variable dense and scarce cellular areas were present in all cases. They consisted of stellated or spindled cells with scant cytoplasm and round nuclei without nucleoli. These cells displayed focal immunoreactivity to smooth muscle actin (Clone 1A4, RTU, Agilent-Dako) and SATB2 (Clone EP281, RTU, Agilent-Dako (Agilent, Frederick, Colorado)), consistent with a bone-forming tumor, with no CD34 (Clone QBEnd-10, RTU, Agilent-Dako) and CD68 (Clone, PG-M1 RTU, Agilent-Dako) positivity. No atypical or pleomorphic cells were observed, and no giant osteoclast-like cells were seen. In two cases, woven bone with no osteoblastic rimming was the predominant component. Newly formed immature woven trabeculae were present in eight cases, pseudopagetoid bone (woven bone with irregular mosaic lines of calcification) was observed in two cases, and a psammomatoid pattern appeared in seven cases. More than one pattern was present in two cases. No cartilaginous areas were seen in our series. Adipose tissue was present in seven cases, found in the periphery in one case and intermixed with other components in the rest of the cases. Two of our three fat signal-detected tumors on MRI were histologically confirmed as adipose tissue. Xantomized cells were detected in seven cases. Microcystic irregular spaces “lymphangioma-like” were observed in six cases and macrocystic spaces in four cases. One case presented ABC-like areas. Macrocystic spaces and ABC-like changes were found in two of our cases where internal septation was detected radiologically. Small hyalinized vessels were seen in 11 cases and large hyalinized vessels in 1 case. Lymphoplasmacytic inflammatory cells were found in seven cases, either forming small groups or dispersed as isolated cells, one with a perivascular pattern. Hemosiderophages were found in two cases. Ischemic necrosis was present in two cases and, in one case, there were bone infarct areas. No malignant transformation was registered in our series. Exemplary histopathological findings are shown in Figure 2 and Figure 3.

Targeted next-generation sequencing was performed on the tumors of two patients, which revealed the presence of a pathogenic missense somatic variant in the *GNAS* oncogene (NM_080425.3) in hotspot position c.2530C > T, p.Arg844Cys (Location 20:57484420, COSM27887; also described as c.601C > T, p.Arg201Cys in the transcript NM_000516.7), with a variant allele frequency of 26% (average coverage in the sample 2556×) and a pathogenic missense variant in the *TP53* tumor suppressor gene (NM_000546.5): c.520A > T, p.Arg174Trp (Location 17:7578410, COSM44782) with a variant allele frequency of 64% (average coverage in the sample 2484×).

The median follow-up spanned 176.4 months (13–312 months). There was no evidence of local recurrence or metastases. Three patients died from other causes (natural death, ovarian tumor, and multi-organ failure). No malignant transformation was detected.

## 4. Discussion

LSMFT was first described by Ragsdale et al. in 1986 [10]. Since then, 241 cases have been reported in the English language literature [2,3,4,5,6,7,10,11,12,13,14,15,16,17,18,19,20,21,22,23,24,25,26,27,28]. LSMFT was not mentioned in the third edition (2002) of the WHO [29] classification of Bone and Soft Tissue Tumors. In the fourth edition (2013), it appears as an osteosarcoma-associated condition, similarly to fibrous dysplasia or bone infarct [30]. In the fifth edition (2020), it does not have its own chapter and is only mentioned as a non-recommended related terminology to fibrous dysplasia [8].

The main characteristics of the previous published cases are summarized in Table 2. The published cases refer to 56 males and 59 females (ratio 1:1.05) with a mean age of 45.8 (from 14 to 84 years old), from the studies where this information was available [2,3,4,5,6,7,10,11,12,13,14,15,16,17,18,19,20,21,22,23,24,25,26,27,28]. In our series, there was a slight predominance in females and a younger mean age.

It was incidentally detected in 47 of the previous published cases [2,3,4,5,6,7,10,11,12,13,14,15,16,17,18,19,20,21,22,23,24,25,26,27,28], with 39 patients initially referred due to local pain and 11 having a pathological fracture. Three patients were referred due to prior local traumatism [15,20]. Most of the cases (149) appeared in the femur, 94.5% of which were located in the intertrochanteric region of the femur [2,3,4,5,6,7,10,11,12,13,14,15,16,17,18,19,20,21,22,23,24,25,26,27,28]. Only four cases were in the distal femur [10,20,24]. Other affected bones included the humerus [2,10] (6), tibia [10,20,26] (4), iliac bone [2,12,20] (5), rib [2] (1), and cranial vault [10,22] (3). All of the cases were solitary lesions (54% of them on the left side), except for two that presented multiple bone lesions [4,14]. In our series, all cases were located in the femur.

In previously reported LSMFTs, plain radiography and CT displayed well-defined, lytic, expansile, and geographic lesions with a sclerotic rim. Internal calcifications were seen in six cases and internal septations in two cases [2,16,17,18,22,23,25,27]. Ground-glass areas appeared in 20 cases [2,3,4,5,6,7,10,11,12,13,14,15,16,17,18,19,20,21,22,23,24,25,26,27,28]. Cystic lesion was observed in one case [6]. Scalloped endosteum was evidenced in two cases [6,15]. There were heterogeneous well-defined masses, hypointense to the skeletal muscle on T1WI and hyperintense on T2WI. They showed heterogeneous enhancement after paramagnetic contrast administration. No distinct fatty components were seen. PET demonstrated intense uptake in the cases where it was performed [16,19,25]. The radiological findings of LSMFT in our patient cohort presented similarities, when compared to previously reported cases. As in prior reports, our plain radiography and CT findings showed LSMFT’s characteristic well-defined lytic, expansile, and geographic lesions with a sclerotic rim. These lesions often presented internal septations and areas of calcification, which were also observed in previous studies. Our MRI findings also presented parallels with those from the medical literature. A fatty component was seen in two cases, as typically described in the literature [2].

The previously reported LSMFTs were histologically heterogeneous lesions, with different components, including bone, adipose tissue, xantomized cells, fibromyxoid stroma, and cyst formations [2,3,4,5,6,7,10,11,12,13,14,15,16,17,18,19,20,21,22,23,24,25,26,27,28]. Fibromyxoid stroma was present in all cases, except in three (in which it was fibrous or collagenoid). Woven bone with pagetoid, curvilinear, and trabecular (fibrous dysplasia-like) patterns was mostly present [2,3,4,5,6,7,10,11,12,13,14,15,16,17,18,19,20,21,22,23,24,25,26,27,28]. Fat necrosis was evident in four cases [6,18,24]. Dystrophic calcifications were observed in three cases [13,18,22]. ABC-like areas were found in one case [6]. Scattered lymphocytes were described in one case [15]. Our cases also presented similar histopathological findings as those described in the literature. The most frequent component of our series was a fibromyxoid stroma followed by woven bone and adipose tissue. All components were intermixed. Histological components not previously reported in the literature included microcystic spaces, hyalinized stroma, small hyalinized vessels, and the presence of hemosiderophages and mast cells. Macrocystic spaces and ABC-like changes were found in two of our cases where internal septation was radiologically detected. In two of our three tumors with fat signal on MRI, the adipose tissue was histologically confirmed.

The immunohistochemical profile of LSMFT has not been previously reported in the medical literature. In our series, consistent with a bone-forming tumor, cells adjacent to woven bone—as well as those distanced from it—revealed SATB2 positivity. Smooth muscle actin was positive in a patchy manner with CD34 negative immunoreactivity. *GNAS* p.Arg844 pathogenic variants have been only studied in four of the published cases, being present only in three of them [12,14].

Malignant transformation occurred in 24 of the published cases (9.9%) [2,3,4,5,10,11,20], mainly to osteosarcoma or high-grade sarcoma. It either existed at the time of diagnosis or developed during the follow-up period. No molecular studies were performed in any of these cases. In cases with a malignant component at initial presentation, a cystic osteosarcoma with fibromyxoid areas should be ruled out.

Curettage and bone grafting were the treatments of choice in most published cases, as in our series. Conservative management was preferable in two published cases, and a transtibial amputation and hemiarthroplasty were performed in two cases with malignant transformation [4,20]. The median follow-up time of the non-malignant published cases was 29.3 months, and no evidence of relapse was seen. Two cases with malignant transformation died due to metastases. No malignant transformation nor local relapses occurred in our series.

LSMFT presents a diagnostic challenge due to its clinical, radiological, and histopathological features overlapping with those of several bone tumors. The most subtle differential diagnosis should be made with conventional fibrous dysplasia, as they present overlapping radiological and pathological features. Monostotic fibrous dysplasia is a benign tumor that affects children and young- and middle-aged adults [18]. They are not usually painful. They arise in meta-diaphysis of long bones or the cranio-facial area and are lytic and geographic non-aggressive lesions with a “ground-glass” appearance on radiological images but lack the fatty and myxoid components of LSMFTs [18]. They may also present a sclerotic rim.

Histologically, fibrous dysplasia consists of a fibrous stroma with bland fibroblastic cells and irregular woven bone trabeculae lacking conspicuous osteoblastic rimming [8]. The percentage of the bone component of reported LSMFT cases is not clearly described. Bone areas were present in less than 10% of the tumor in more than half of our cases. Diagnostic difficulty could arise if the heterogenicity of LSMFT is not known. On rare occasions, fibrous dysplasia may contain multi-nucleated osteoclast-like giant cells, myxoid areas, metaplastic fat areas, or xantomized cells. No small hyalinized vessels have been described in fibrous dysplasia [8]. Some authors have proposed that LSMFT may be a traumatized fibrous dysplasia variant based on the fact that the proximal femur is more susceptible to fatigue fracture [6]. While this is an interesting point of view, prior local traumatism was present only in 0.02% of all cases, which leads us to believe that it is probably a non-related condition. Four of our cases were initially diagnosed as fibrous dysplasia and re-classified as LSMFT based on the presence of any of the following: little bone areas, large adipocytic areas, large fibromyxoid areas, and/or small hyalinized vessels.

Fibrous dysplasia has been shown to harbor a missense mutation at codon 201 in exon 8 of GNAS [12]. GNAS p.Arg844 pathogenic variants have been previously described in LSMFT [12,14], but GNAS mutations have been studied only in 0.02% of all LSMFT cases. It is well known that the same genetic mutation may present different phenotypes. GNAS-activating mutations have been identified across many cancer types, such as pancreatic adenocarcinoma, squamous cell carcinoma of the head and neck, breast neoplasm, gastric adenocarcinoma, adrenal cortex carcinoma, and sex cord-stromal tumor, as well as in fibrous dysplasia and LSMFT. Nevertheless, the precise role of this mutation in tumor development or in malignant transformation in LSMFT cases remains unknown. The GNAS oncogene, which is known to be implicated in cell proliferation and tumoral invasiveness, could be considered as a new prognostic factor for these tumors. TP53—which encodes tumor suppressor p53—is the most frequently mutated gene in human cancers. TP53 mutations are also potentially prognostic and predictive markers, as well as targets for pharmacological intervention [31]. To date, however, no variants in this gene have been described in LSMFT, and no TP53 mutations have been described in fibrous dysplasia [8]. Molecular studies in large cohorts of patients are required to elucidate the roles of these two genes in LSMFT.

Other benign tumors are mistaken in the differential diagnosis of LSMFT due to its polymorphic appearance, although most of these tumors do not usually show radiological similarities. Chondromyxoid fibromas are benign tumors that can affect any bone of young adults [7]. They are eccentric, well-defined lytic lesions that may contain small calcifications on radiological studies. They consist of lobules with central chondromyxoid areas and peripheric multi-nucleated osteoclast-like giant cells. *GMR1* mutations have been found in this type of tumor. Enchondromas are benign chondrogenic tumors that arise at any age in the meta-diaphysis of short and long tubular bones [18]. They are well-defined radiolucent and lytic neoplasms with central punctate “popcorn” calcifications. They do not, however, present the mixed lytic and sclerotic pattern of LSMFTs [18]. They are histologically composed of small and separated hypocellular hyaline cartilage nodules with a metaplastic bone rim and small chondrocytes in their lacunae [18]. Cartilage areas are exceptional in LSMFT, having only been described in a case series [28].

Osteoblastomas are locally aggressive bone formation tumors that tend to develop over the second and third decades of life [20]. Although any bone may be affected, they typically arise in posterior vertebrae elements. They measure over 2 cm and are clearly lytic masses with reactive peripheral sclerosis and central mineralization [20]. Anastomosing trabecular rimmed by osteoblasts and scattered multi-nucleated osteoclast-like giant cells are commonly histologically observed [20], and *FOS* translocations are usually present. Intraosseous lipomas may appear at any age and in any bone [2]. They are lytic masses with a sclerotic rim and evidence of homogeneous macroscopic fat through CT or MRI [1]. They consist of mature adipocytic tissue, and areas of fat necrosis may be present [1]. Bone infarcts may appear at any age and in any bone [18]. They are lytic lesions with central lucency surrounded by sclerosis with a serpiginous border imitating “smoke up the chimney” [18]. Necrotic bone areas, dystrophic calcifications, fat necrosis, or hyaline fibrous tissue may be histologically seen [18], as in LSMFT.

Other cystic bone lesions should be considered in those LSMFT cases with an evident cystic component. Simple bone cysts appear during the first decade of life. They may be asymptomatic or can cause pathological fractures [18]. They are more frequently found in humeral and femoral meta-diaphysis [18]. They are well-defined lytic masses with cortical thinning and a sclerotic rim. The “fallen fragment” radiological characteristic sign is seen in this lesion [18]. It consists of thin, unicystic walls filled with clear, serous liquid [18]. ABCs are usually eccentrically located expansile lesions with a multi-chamber appearance, with multiple septations and low signals on T1 and T2 that demarcate spaces with fluid–fluid levels and demonstrate enhancement after contrast administration, resulting in a “honeycomb appearance”, in contrast with LSMFTs [2]. In histological terms, they are blood-filled cystic spaces without an endothelial lining separated by thin septa. The fibrous septa contain a dense proliferation of fibroblasts, scattered multi-nucleated osteoclast-type giant cells, and strands of woven bone rimmed with osteoblasts [32]. Intraosseous ganglion cysts are usually found around the ankle [6]. Radiologically, they are lytic, multi-loculated lesions with a sclerotic rim and a fluid-like appearance, with fibrous tissue with no epithelial cell lining and translucent gelatinous material seen upon pathological examination.

## 5. Conclusions

LSMFTs should be included in the differential diagnosis of solitary bone lesions in the femoral intertrochanteric region. It is important to recognize typical radiological findings and heterogeneous histopathological components, especially in small biopsies, in order to avoid the misdiagnosis or overtreatment of patients. Keeping in mind that up to approximately 10% of the considered cases had a reported malignant transformation is key to providing accurate monitoring of potentially affected patients.

## Figures and Tables

**Table 1:** Main characteristics of our LSFMT cases.

**Case**	**Age, Sex**	**Location**				**Symptoms**	**Radiology**			Histology											Molecular Tests	Treatment	Evolution
		**Side**	**Bone**	**Site**			**X-Ray/CT**	**MRI/PET/SPECT**	**Stroma**	**Cells**	Bone	Adipose	Xantomized Cells	Cystic Spaces		Vessels	Inflammation	Necrosis	Other	Malignant Transformation			
														Micro	Macro								
1	M, 26	R	Femur	P	IT	Pathological fracture	Ly, Sc rim	INA	95% Fibromixoid	Stellated, spindled No atipia	3% Psamomatoid	No	No	2%	No	Small, hyalinized	No	No	-	No	Not performed	Curettage and bone grafting	312 months, NED
2	F, 24	R	Femur	P	IT	ID	INA	INA	75% Fibromixoid	Stellated, spindled No atipia	4% Psamomatoid	15% Peripheral	1%	5%	No	Small, hyalinized	Mast cells	No	-	No	Not performed	Curettage and bone grafting	215 months, NED
3	M, 61	R	Femur	P	IT	ID (Hodgkin lymphoma extension study)	INA	INA	20% Fibromixoid	Stellated, spindled No atipia	40% Gross trabeculae, Pagatoid	40% Intermixed	No	No	No	Small, hyalinized	Mast cells, lymphocytes	No	-	No	Not performed	Curettage and bone grafting	192 months, NED Death (multi-organ failure)
4	M, 19	L	Femur	D	-	Pain	Ly, cystic, internal trabeculae	Ly, cystic, internal trabeculae T2WI: HI Fat areas	80% Hyalinized	Stellated, spindled No atipia	3% Psamomatoid	10% Intermixed	6%	No	1%	Small, hyalinized	No	No	-	No	Not performed	Curettage and bone grafting, nail-plate	281 months, NED
5	M, 28	L	Femur	P	IT	ID	Ly, Sc rim	Ly, Sc rim T1WI: isointense T2WI: HI	95% Fibromixoid	Stellated, spindled No atipia	2% Trabeculae	No	3%	No	No	Small, hyalinized	Mast cells	No	-	No	Not performed	Curettage and bone grafting, nail-plate	145 months, NED
6 External case	F, 48	R	Femur	P	IT	Pain	Ly, cystic, internal trabeculae	INA	75% Fibromixoid	Stellated, spindled No atipia	10% Trabeculae	2% Intermixed	No	No	3%	Small, hyalinized	No	10% Ischemic	ABC –like areas	No	Not performed	Curettage and bone grafting	303 months, NED
7	F, 20	L	Femur	P	IT	Pain Previous local trauma	INA	INA	95% Fibrous	Stellated, spindled No atipia	2% Psamomatoid	No	1%	3%	No	Small, hyalinized	No	No	-	No	Not performed	Curettage and bone grafting, nail-plate	134 months, NED
8	M, 68	R	Femur	P	IT	ID	Ly, Sc rim	Sc rim, internal septae PET: no uptake	70% Fibromixoid	Stellated, spindled No atipia	30% * Trabeculae * CNB	No	No	No	No	Not seen	Plasma cells	No	-	No	Not performed	Curettage and bone grafting	167 months, NED
9	M, 19	R	Femur	P	IT	Pain	WD, Ly, Sc rim	Bone scintigraphy and PET: peripheral uptake	77% Mixoid > fibro	Stellated, spindled No atipia	3% Trabeculae	No	No	15%	5%	Small, hyalinized	Lymphocytes, hemosiderophages	1% Ischemic	-	No	NGS: TP53 mutation (c.520A > T, p.Arg174Trp)	Curettage and bone grafting, nail plate	38 months, NED
10	F, 50	L	Femur	P	IT	ID (ovarian tumor extension study)	WD, Ly, Sc rim	T1WI: hi T2WI: HI SPECT: moderate homogeneous uptake	50% Fibromixoid	Stellated, spindled No atipia	50% * Trabeculae * CNB	No	No	No	No	Not seen	No	No	-	No	Not performed	Expectant management	13 months, NED Death (ovarian tumor)
11	F, 52	L	Femur	P	IT	Pain	WD, Ly, Sc rim	T1–WI: hi T2–WI: HI Fat areas	90% Fibromixoid, dense collagen bands	Stellated, spindled No atipia	5% Psamomatoid	No	2%	No	3%	Small, hyalinized	Lymphocytes	No	-	No	NGS: GNAS mutation (c.2530C > T, p.Arg844Cys )	Curettage and bone grafting	41 months, NED
12 External case	F, 72	L	Femur	P	IT	Pain	WD, Ly, Sc rim	INA	94% Fibromyxoid	Stellated, spindled No atipia	5% Psamomatoid	No	No	1%	No	Not seen	No	No	-	No	Not performed	Curettage and bone grafting	240 months, NED Natural death
13	M, 38	R	Femur	P	IT	Pathological fracture	INA	T1WI: hi T2WI: HI Fat areas	25% Fibromyxoid	Stellated, spindled No atipia	30% Pagetoid	10% Intermixed	5%	No	No	Small, hyalinized	No	No	Bone infarct (30%)	No	Not performed	Curettage and bone grafting	257 months, NED
14 External case	F, 26	L	Femur	P	IT	ID	INA	INA	72% Fibromyxoid	Stellated, spindled No atipia	15% Ttrabeculae, pagatoid, psamomatoid	1% Intermixed	4%	8%	No	Small, hyalinized	Perivascular plasma cells and lymphocytes	No	-	No	Not performed	Curettage and bone grafting, nail plate	129 months, NED
15	M, 18	R	Femur	P	IT	Pain	INA	INA	60% Mixoid > fibro	Stellated, spindled No atipia	30% Trabeculae	10% Intermixed	No	No	No	Big, hyalinized	Hemosiderophages	No	-	No	Not performed	Curettage and bone grafting	131 months, NED

R: right; L: left; P: proximal; D: distal; IT: intertrochanteric; ID: incidentally detected; WD: well-defined; Ly: lytic; Sc: sclerotic; HI: hyperintense; WI: weighted images; CT: computed tomography; MRI: magnetic resonance imaging; PET: positron emission tomography; SPECT: single-photon emission computed tomography, INA: information not available; CNB: core needle biopsy; ABC: aneurysmal bone cyst. NED: no evidence of disease; NGS: next-generation sequencing.

**Table 2:** Summarized characteristics of previous published cases of LSFMT.

**Authors, Year**	**Nº of Cases**	**Age, Sex**	**Location**				**Symptoms**	Radiology		Histology							Treatment	Evolution
			**Side**	**Bone**	**Site**			**X-Ray/CT**	MRI/PET/SPECT	Stroma/Cells	Bone	Adipose	Xantom. Cells	Cystic Spaces	Other	Malignant Transformation		
Ragsdale et al., 1993 [28]	95	21–67 (mean 43)	INA	Femur (87) Tibia (2) Humerus (4) Cranial vault (2)	Femur: P (85), D (2)	INA	ID, pain, pathological fracture	WD, Ly, Sc rim, increase uptake	INA	INA	WB, LB Pagetoid	Yes	Yes	INA	Cartilage areas (rare)	Yes (15)	INA	INA
Gilkey et al., 1993 [11]	40	INA	INA	INA	INA	INA	INA	INA	INA	INA						Yes (1)	INA	INA
Krandsorf et al., 1999 [2]	39	15–69 (mean 42) M (21), F (18)	INA	Femur (33) Iliac bone (3), humerus (2) Rib (1)	Femur: P (30), shaft (3)	IT (30)	Pain (14), ID (12), pathological fracture (3)	Ly, WD, geographic, variable Sc areas, variable mineralized matrix	Well-defined, variable-thickness peripheral rim, T1WI: isointense, homogeneous, T2WI: HI, heterogeneous, STIR: HI	INA						Yes (4): osteosarcoma (2), malignant fibrous hystiocitoma (1), low grade malignant change (1)	INA	INA
Heim-Hall et al., 2003 [6]	4	14, M	R	Femur	P	ST	Pathological fracture	Ly, ground-glass opacity, scalloped endosteum	INA	Fibromyxoid	Trabecular (FD-like)	Fat necrosis	Yes	No	-	No	Curettage	INA
		74, F	L	Femur	P	IT, ST	Pain	Cystic lesion, Sc margins	INA	Fibromyxoid	Pagetoid Bone necrosis	Yes	Yes	No	-	No	Curettage and grafting	INA
		37, F	L	Femur	-	-	Pain	WD, lucent, thick bony borders	INA	Fibromyxoid	Trabecular (FD-like)	Yes	Yes	No	-	No	Curettage	INA
		37, F	R	Femur	-	-	Pathological fracture	Ly, ground-glass opacity, scalloped endosteum	INA	Fibromyxoid	Trabecular (FD-like)	Fat necrosis	No	No	ABC component	No	Curettage and grafting	INA
Matsuba et al., 2003 [12]	2	75, M	R	Femur	P	IT	Pain	WD, Ly, Sc rim	T2WI: hi and HI	Fibromyxoid	Curvilinear, trabecular (FD-like)	Yes	Yes	No	GNAS mutation	No	Curettage and grafting	16 months, NED
		59, M	INA	Iliac bone	-	-	ID	WD, Ly, Sc rim	T2WI: hi and HI	Fibromyxoid	Curvilinear, trabecular (FD-like)	Yes	Yes	No	GNAS mutation	No	Curettage and grafting	24 months, NED
O’Dwyer et al., 2005 [13]	1	51, M	L	Femur	P	IT	Pain	WD, Ly, Sc rim	T1WI: hi T2WI: HI STIR: HI	Fibromyxoid	WB Trabecular (FD-like), pagetoid, spicules	Yes	Yes	No	Dystrophic calcification	No	INA	INA
Choi et al., 2005 [26]	1	61, M	L	Tibia	P	Diaphysis	ID	WD, Ly, Sc rim	T1WI: hi T2WI: HI Heterogeneous enhancement	Fibromyxoid	WB Trabecular (FD-like), pagetoid	Yes	Yes	No	-	No	Curettage	INA
Corsi et al., 2006 [14]	2	33, F	L	Femur	P	Neck	Multiple bone lesions: monostotic FD	Ground-glass lesion, increased uptake	INA	INA	Trabecular (FD-like)	Yes	Yes	Yes	No GNAS mutation detected	No	Curettage	INA
		54, F	L	Femur	P	IT	ID	Lucent lesion with scattered densities and a Sc rim	INA	Fibromyxoid	Trabecular woven bone	No	Yes	No	GNAS mutation	No	Curettage and grafting	INA
Rao et al., 2008 [15]	1	35, F	L	Femur	P	IT	Pain, prior local trauma	WD, Ly, ground-glass opacity, scalloped endosteum	INA	Fibromyxoid	Spicules	No	Yes	No	Scattered lymphocytes	No	INA	INA
Campbell et al., 2008 [3]	1	42, F	L	Femur	P	IT	Pain and pathological fracture	WD, Ly, Sc rim	INA	Fibromyxoid	INA	Yes	Yes	No	-	Yes, OS with giant cells	Curettage and cemented fixation	31 months, death (lung metastases)
Teruel-González et al., 2008 [27]	1	43, M	L	Femur	P	IT	ID	WD, Ly, internal Ca	T1WI: hi T2WI: HI	Fibromyxoid	WB Pagetoid, trabecular (FD-like)	Yes	Yes	No	-	No	Curettage and grafting	NED
Nieto et al., 2009 [16]	2	50, M	R	Femur	P	IT	ID (femoral chondrosarcoma extension study)	WD, Sc rim	PET: increased activity	Fibromyxoid	Yes	No	Yes	No	-	No	INA	INA
		57, M	R	Femur	P	IT	Pain	WD, Ly, Sc rim, internal Ca	T1WI: hi T2-WI: HI	INA							INA	INA
Dattilo et al., 2012 [7]	33	19–80 (mean 46) M (20), F (13)	INA	Femur	P	IT	ID (21) Pain (10) Pathological fracture (2)	WD, geographic lucencies, Sc rim Ground-glass areas (15)	T2WI: heterogeneous	Fibromyxoid	Reactive bone, woven bone	Yes (3)	No	No	-	No	Curettage and grafting (13)	INA
Illac et al., 2012 [5]	1	84	L	Femur	P	IT	Pain Multiple bone lesions	WD, Ly, hipodense	T2WI: HI Heterogeneous enhancement	INA	INA	INA	Yes	INA	-	Yes, TG OS	INA	INA
González et al., 2014 [17]	1	25, M	L	Femur	P	IT	Pain	WD, Ly, Sc rim, internal Ca	INA	Fibromixoid	Trabecular (FD-like)	Yes	Yes	No	-	No	INA	INA
Técualt-Gómez et al., 2015 [18]	1	80, M	R	Femur	P	IT	Pain	WD, Ly, Sc rim, internal Ca	T1WI: hi T2WI: HI	Fibromixoid	Trabecular (FD-like) Bone necrosis	Fat Necrosis	No	No	Dystrophic calcification	No	Curettage and PMMA	INA
Kim et al., 2015 [19]	1	22, F	R	Femur	P	INA	ID (Hodgkin lymphoma extension study)	WD, Ly, Sc rim	PET: intense uptake	Fibromixoid	Trabecular	No	No	No	-	No	INA	INA
Regado et al., 2016 [20]	9	50, F	R	Femur	P	IT	Pain and limping Local edema (2) Local trauma (2) Pathological fracture (3)	WD, Ly, Sc rim	INA	Fibromixoid Stellate, fusiform, bipolar and oval cells	Bone necrosis	Yes	Yes	Yes	-	Yes (1) Increased cellularity and atypical nuclei	Curettage and grafting	14 months, NED
		53, M	R	Tibia	D	-		WD, Ly, Sc rim, cortical disruption									Transtibial amputation	97 months, NED
		19, M	R	Femur	D	-		WD, Ly, Sc rim									Curettage and grafting	76 months, NED
		45, F	R	Femur	P	IT		WD, Ly, Sc rim									Curettage and grafting	66 months, NED
		42, F	L	Femur	P	IT		WD, Sc									Curettage and grafting	66 months, NED
		50, F	L	Iliac bone	-	-		WD, Ly, Sc rim									No treatment	40 months, NED
		39, F	R	Femur	P	IT		WD, Ly, Sc rim									Curettage and grafting	29 months, NED
		30, F	R	Femur	P	IT		WD, Ly, Sc rim									Curettage and grafting	28 months, NED
		27, F	L	Femur	P	IT		WD, Ly, Sc rim									Curettage and grafting	14 months, NED
Beytemür et al., 2017 [25]	1	67, M	R	Femur	P	IT	Pain	WD, Sc rim, internal septations	T1WI: hi T2WI: HI Heterogeneous enhancement PET: increased activity	Fibromixoid	Trabecular (FD-like)	No	Yes	No	-	No	Curettage	16 months, NED
Kampen et al., 2018 [21]	1	69, M	L	Iliac bone	-	-	Pain	WD, Sc rim	T2WI: HI Slight Enhancement SPECT/CT: EP: no abnormal hyperperfusion DP: increased activity	Fibrous	WB, LB Curvilinear, pagetoid	No	Yes	No	-	No	INA	INA
Ploof et al., 2019 [22]	1	30, F	L	Cranial vault	-	-	Slow growing painless mass	WD, expansile, scalloped endosteum, scattered peripheral Ca	Heterogeneous enhancement	Fibromixoid	WB Curvilinear	Yes	No	Yes	Dystrophic calcification	No	Complete excision	8 months, NED
Bahk et al., 2020 [4]	1	75, F	R	Femur	P	IT	Multiple bone lesions: polyostotic FD, AVN (left femur)	Lobular, ground-glass opacity	Heterogeneous T1WI: hi T2WI: HI	Fibrous	WB Pagetoid, spicules	Yes	No	No	-	Yes, high grade sarcoma (FS)	Bipolar hemiarthroplasty with cementation	5 months, death (multiple metastases)
Pai et al., 2021 [23]	1	63, M	L	Femur	P	IT, ST	ID	WD, Ly, Sc rim, internal septations	Heterogeneous T1WI: hi T2WI: HI	INA						No	Conservative management	6 months, NED
Zhang et al., 2022 [24]	1	55, F	L	Femur	D	-	Pain	Patchy high-density shadow	Heterogeneous T1WI: hi T2WI: HI	Collagenoid, myxoid degeneration	Yes	Fat necrosis	No	Yes	-	No	Curettage and bone grafting	6 months, NED

Sex: F: female; M: male. Side: L: left; R: right. Site: P: proximal; D: distal. Bone site: IT: intertrochanteric; ST: subtrochanteric. Symptoms: ID: incidentally detected; FD: fibrous dysplasia; AVN: avascular necrosis. Radiology: CT: computerized tomography; MRI: magnetic resonance image; PET: positron emission tomography; SPECT: single-photon emission computed tomography; WI: weighted images; STIR: short tau inversion recovery sequences, WD: well-defined; Ly: lytic, Sc: sclerotic; HI: hyperintense. hi: hypointense; EP: early phase; DP: delayed phase; ABC: aneurysmal bone cyst. Histology: WB: woven bone; LB: lamellar bone; OS: osteosarcoma; TG OS: telangiectatic osteosarcoma; FS: fibrosarcoma. Treatment: PMMA: polymethylmethacrylate; NED: no evidence of disease; INA: information not available.

## Data Availability

Research data are available upon reasonable request to the authors.
